# Extracellular Vesicles Derived from Natural Biological Resources and Their Potential to Facilitate Skin Regeneration and Rejuvenation

**DOI:** 10.3390/pharmaceutics18030342

**Published:** 2026-03-10

**Authors:** Zhuoyue Yang, Shijun Li, Hangyu Zhang, Zhigang Sui, Na Li

**Affiliations:** 1 Central Hospital of Dalian University of Technology, Dalian 116033, China; yangzhuoyue6@163.com (Z.Y.); lshijun@126.com (S.L.); 2 Faculty of Medicine, Dalian University of Technology, Dalian 116024, China; hangyuz@dlut.edu.cn; 3 Liaoning Key Laboratory of Integrated Circuit and Biomedical Electronic System, Dalian University of Technology, Dalian 116024, China; 4 Key Laboratory of Separation Science for Analytical Chemistry, National Chromatographic R&A Center, Dalian Institute of Chemical Physics, Chinese Academy of Sciences, Dalian 116023, China

**Keywords:** tissue-derived extracellular vesicles, plant-derived extracellular vesicles, wound healing, skin photoaging, skin regeneration

## Abstract

The skin, the largest organ in the human body, serves as a crucial barrier against external stimuli. With the acceleration of social industrialization and the worsening of global climate change, the risk of physical, chemical and biological damage to the skin has significantly increased. Among these, surgical wounds, accidental injuries, diabetic wounds, and ultraviolet (UV)-radiation-induced photoaging are particularly common. Cutaneous wound healing is a complex and dynamic process that requires precise coordination of numerous molecular events to effectively repair damaged skin. Skin photoaging, a phenomenon of premature aging caused by long-term UV exposure, is characterized by pigmentary abnormalities, telangiectasia, epidermal roughness, wrinkle formation, and precancerous lesions, all of which seriously affect skin health and appearance. Extracellular vesicles (EVs), a class of nano-sized vesicles secreted by various cells, play important regulatory roles in tissue regeneration. Although cell-culture-medium-derived EVs (C-EVs) have been proven to effectively promote skin wound healing and photodamage repair, their origin from a single cell type and challenges in large-scale production severely limit their broad application. In contrast, EVs derived from natural biological resources, including tissue-derived EVs (Ti-EVs) and plant-derived EVs (PDEVs), have emerged as novel therapeutic strategies for skin wounds and photoaging. These EVs better reflect the physiological microenvironment and demonstrate considerably higher production efficiencies. Ti-EVs, obtained from mammalian tissues composed of multiple cell types and extracellular matrix, contain more abundant regulatory factors, thus exhibiting superior bioactivity compared with C-EVs. PDEVs have also garnered significant attention due to their favorable stability, low immunogenicity, unique natural antioxidant components, and feasibility of large-scale extraction. This review will systematically elaborate on the characteristics and isolation methods of both Ti-EVs and PDEVs, as well as their therapeutic roles and underlying mechanism in wound healing and skin photoaging.

## 1. Introduction

As the largest organ of the human body, the skin serves as an effective barrier against pathogen invasion and represents the first line of immune defense [1,2]. This role leads to direct contact of the skin with diverse extrinsic stimuli and frequent damage. Among various damaging factors, two are particularly prominent: trauma-induced wounds and ultraviolet (UV)-radiation-induced photoaging, both of which pose serious challenges to skin integrity and function recovery, attracting significant interest in the fields of skin regeneration and rejuvenation. Wound healing is a complex process that is often delayed in patients with underlying chronic diseases. The cost of managing non-healing wounds places a heavy burden on society, creating an urgent need for innovative therapeutic strategies to promote healing. Photoaging accelerates skin aging and raises the risk of apoptosis and carcinogenesis through irreversible collagen degradation, DNA damage and a series of inflammatory reactions.

Recently, cell-free therapy based on extracellular vesicle (EV) therapy has gained significant interest due to its low heterogeneity, high biocompatibility and excellent capacity to cross biological barriers [3,4]. EVs are nanoscale particles secreted by virtually all cell types and are abundantly present in tissue fluids. They play a key role in intercellular communication under physiological and pathological conditions by transferring bioactive molecules, including protein, lipids, and nucleic acids. EVs have been demonstrated to serve as both diagnostic markers and therapeutic agents for various diseases, such as cardiovascular disorders, skin wounds and photoaging. Despite the growing recognition of cell-culture-medium-derived EVs (C-EVs) in promoting cutaneous wound healing and mitigating photoaging, while also avoiding the safety concerns associated with cell-based therapies, their application remains constrained by challenges including low yield, high cost and time consumption, primarily due to the need for large volumes of cell culture supernatant. Moreover, the artificial conditions of in vitro cell culture differ substantially from the native physiological environment. As a result, EVs secreted by a single cell type may not fully recapitulate the complex, multicellular nature of tissue microenvironments. Consequently, neither homogeneous cultured cell lines nor primary cell-derived EVs can entirely capture the local molecular signatures of the physiological or pathological tissues from which they originated.

In contrast, EVs derived from natural resources, including mammalian-tissue-derived EVs (Ti-EVs) and plant-derived extracellular vesicles (PDEVs), not only offer the benefits of containing multicellular bioactive components and enabling high-yield production but also demonstrate enhanced capabilities in promoting tissue regeneration and organ repair through multiple molecular targets. Ti-EVs, which naturally exist in the interstitial space surrounding tissue cells, have been successfully isolated from human adipose tissue, skin, tumors, etc., and these vesicles have been extensively reported to act as potent mediators of intercellular communication and facilitate skin regeneration in the context of cutaneous wounds and photoaging. Moreover, the biological activities of PDEVs derived from various fruits, vegetables and traditional Chinese medicines in promoting skin repair have also been increasingly recognized in recent years. Vesicular cargo components (proteins, nucleic acids, lipids, metabolites) are responsible for the therapeutic effects of natural-resource-derived EVs for skin repair. For instance, Ti-EVs carry membrane proteins (e.g., integrins) for uptake, while PDEVs contain unique proteins (e.g., lectins) and lipids (e.g., phytosterols) with enhanced anti-inflammatory and antioxidant effects. Moreover, Ti-EVs and PDEVs usually share analogous therapeutic mechanisms for the same bioactivity. EV tissue targeting and delivery are regulated by surface molecules (e.g., integrins on mammalian EVs, lectins on plant EVs for skin targeting), cargo composition, the target tissue microenvironment, and physical properties (small EVs have better penetration) [5,6,7].

Herein, we systematically review recent studies on EVs derived from natural resources. We focus on the restorative and regenerative potential of Ti-EVs and PDEVs in the context of both cutaneous wound healing and UV-radiation-induced skin photoaging. This review also discusses the underlying mechanisms and future perspectives, aiming to provide new insights into the therapeutic application of natural-resource-derived EVs in skin repair.

## 2. Skin Wound Healing and Photoaging

The skin, with its three distinct layers (epidermis, dermis, and subcutaneous tissue), constitutes the body’s line of defense, performing critical physiological functions to ward off physicochemical damage and sustain internal homeostasis [8,9,10]. Its unique position as an interface with the external environment, however, predisposes it to a spectrum of injuries that compromise its barrier integrity and lead to pathological conditions. Currently, the processes of wound healing and photoaging represent two of the most significant areas of investigation within cutaneous pathophysiology.

### 2.1. Wound Healing

**Figure 1:**
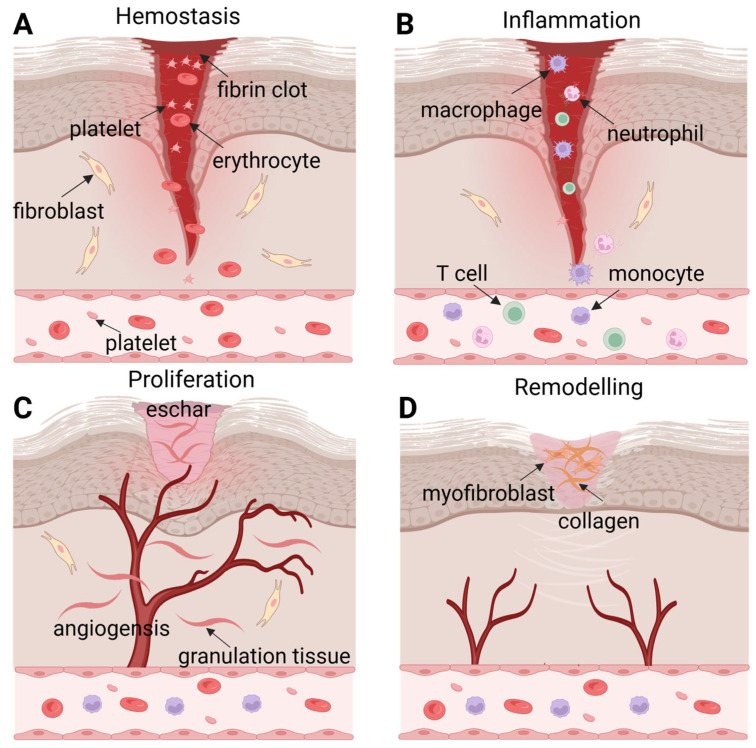
The four stages of wound healing: The process starts with hemostasis, which is characterized by platelet aggregation and the formation of a fibrin clot to minimize bleeding (**A**). Hemostasis is followed by the inflammatory phase, which involves the recruitment of innate immune cells (neutrophils, macrophages) and adaptive immune cells (T cells) to debride the wound and combat pathogens (**B**). The proliferation phase is marked by angiogenesis, the formation of granulation tissue, fibroblast migration, and re-epithelialization, leading to eschar formation (**C**). Finally, the remodeling phase commences, during which myofibroblasts contract the wound and collagen is deposited and restructured to regain tensile strength (**D**). Created in BioRender. Yang, Zhuoyue. (2026) https://BioRender.com/rvchgkg (accessed on 4 March 2026).

Wounds, defined as a persistent disruption of skin structure, typically trigger a series of localized and systemic physiological and pathological alterations [11]. These changes not only impose substantial socioeconomic burdens but also severely compromise patients’ physical and mental well-being. Wound healing is a complex and dynamic process that proceeds through four overlapping yet interdependent phases: hemostasis, inflammation, proliferation, and tissue remodeling [12]. This intricate process requires a cascade of synchronized cellular events to restore damaged skin, including cell migration, proliferation, differentiation, angiogenesis, extracellular matrix (ECM) deposition, and tissue remodeling (Figure 1).

The wound healing process is initiated by hemostasis, during which local blood vessels constrict and platelets aggregate to form a fibrin clot, thereby inducing coagulation and minimizing blood loss [13]. The fibrin clot typically fills the injury site within 12 to 24 h post-wounding, while also creating a protective barrier that prevents fluid loss and pathogen invasion. Following hemostasis, the inflammatory phase is triggered by the infiltration of external pathogens and mediated by inflammatory cells, including neutrophils, macrophages, monocytes, and lymphocytes. These cells are recruited to the wound site, where they interact with cytokines to cause an inflammatory response and defend the host [14,15]. Concurrently, secreted cytokines promote subsequent cell proliferation at the injury site [16]. Therefore, appropriate regulation of the inflammatory response promotes efficient wound healing, whereas an aberrant inflammatory phase may lead to abnormal healing outcomes, such as depressed or hypertrophic scars. The proliferative phase encompasses three key events: angiogenesis, granulation tissue formation and re-epithelialization. Macrophages and injured endothelial cells release fibroblast growth factor-2 (FGF-2) and vascular endothelial growth factor (VEGF) to promote angiogenesis, which is a critical step during wound repair. Granulation tissue serves as a transient reparative structure, with its formation involving multiple cytokines, growth factors, and diverse cell types. Eventually, the granulation tissue is replaced by normal connective tissue during the remodeling phase [17,18]. Re-epithelialization occurs as epithelial cells migrate from the edge to the injured area, ultimately covering the wound and re-establishing an intact epithelial barrier. The final stage of wound healing is tissue remodeling, which typically begins 2 to 3 weeks after injury and may persist for years. During this stage, collagen undergoes continuous degradation and the ECM is restructured [19]. Type III collagen is gradually degraded, while synthesis of type I collagen increases, restoring the tensile strength of the skin. Additionally, most blood vessels, fibroblasts, and inflammatory cells disappear from the wound area as remodeling progresses [20].

### 2.2. Skin Photoaging

Skin aging is the result of intrinsic and extrinsic factors. Intrinsic aging is driven by genetically programmed processes such as telomere shortening, alongside the cumulative effects of oxidative stress and the formation of advanced glycation end products [21,22,23,24]. Among extrinsic factors, long-term exposure to UV radiation is well recognized as the primary driver of skin photoaging. Different from intrinsic aging, photoaging represents a phenomenon of premature aging caused by UV exposure. It is clinically and histologically characterized by an accelerated decline in both the structure and function of the skin [25].

Owing to atmospheric ozone layer filtration, the UV wavelength that can eventually be exposed to the human body is between 290 and 400 nm. UVB (290–320 nm) that is mainly absorbed by keratinocytes in the epidermis has a short wavelength, high frequency and strong energy; thus, its long-term radiation induces skin thinning, abnormal thickening of the stratum corneum, and increased melanin content. Although the energy of UVA (320–400 nm) is lower than that of UVB, its content in the earth’s atmosphere is approximately 20 times that of UVB, leading to its more profound and complex effect. UVA penetrates into the deep dermis, where it degrades collagen and elastic fibers, causing skin laxity and wrinkles, and promotes melanin deposition, leading to hyperpigmentation [26]. Furthermore, long-term UV radiation not only induces irreversible damage to biological macromolecules, such as DNA, RNA and proteins, but also leads to the accumulation of deleterious alterations in skin cells and an increased risk of apoptosis and carcinogenesis [27].

**Figure 2:**
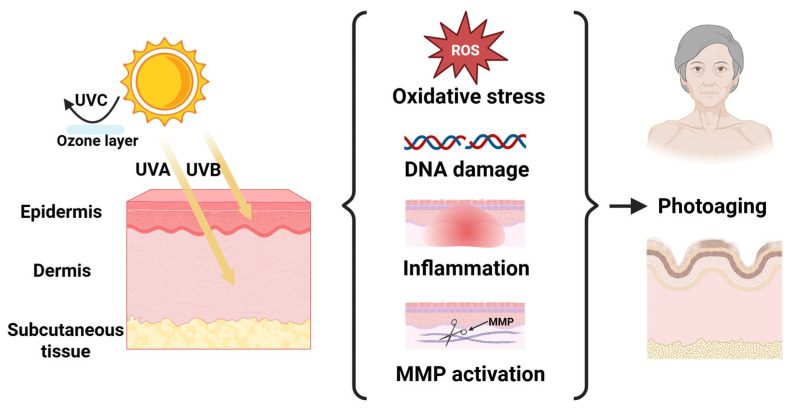
The mechanism of photoaging: Ultraviolet A (UVA) and B (UVB) radiation causes damage to dermal and epidermal cells, while UVC is absorbed by the ozone layer. UV radiation induces the production of reactive oxygen species (ROS), leading to DNA damage, activation of inflammatory pathways, and the production of matrix metalloproteinase (MMP). Created in BioRender. Yang, Zhuoyue. (2026) https://BioRender.com/7pzy10r (accessed on 4 March 2026).

UV-induced cutaneous photoaging is driven by four interconnected pathological mechanisms: oxidative stress, DNA damage, inflammatory cascades, and matrix metalloproteinase (MMP) activation (Figure 2). Among these, oxidative stress serves as the primary pathological driver, mediating lipid peroxidation, protein denaturation, and genomic instability through reactive oxygen species (ROS) accumulation coupled with antioxidant enzyme suppression [28]. DNA damage constitutes another vital mechanism underlying UV-induced skin aging and carcinogenesis. Given that stable cell cycle arrest is a defining feature of cellular senescence, DNA damage causes such an arrest by distorting DNA structure as well as interfering with normal replication and transcription processes [25,29]. UV-induced skin inflammation is closely linked to the accumulation of ROS, which activates the mitogen-activated protein kinase (MAPK) signaling pathway and initiates the transcription factors activator protein 1 (AP-1) as well as nuclear factor kappa B (NF-κB) to upregulate pro-inflammatory factors, including interleukin-1 (IL-1) and tumor necrosis factor alpha (TNF-α) [26]. Furthermore, ROS potentiates MMP activation during photoaging, thereby driving collagenolysis and ultimately compromising dermal fibroblast function.

## 3. EVs

According to the International Society of Extracellular Vesicles (ISEVs), EVs are defined as nanoscale particles enclosed by a lipid bilayer membrane and released by cells, which have no ability of autonomous replication and mediate intercellular communication by carrying bioactive molecules [30]. In recent years, EVs have emerged as a promising strategy for disease diagnosis and therapy, owing to their inherent low immunogenicity, high biocompatibility, and superior capacity for traversing biological barriers.

### 3.1. Biogenesis of EVs

**Figure 3:**
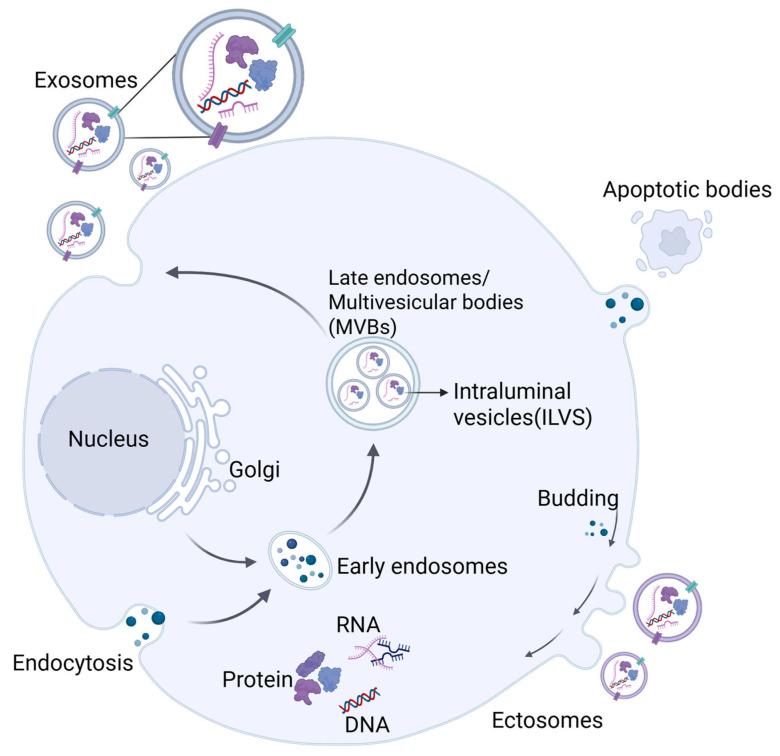
Biogenesis pathways of extracellular vesicles (EVs). Exosomes (30–150 nm) originate from the endosomal pathway: early endosomes mature into late endosomes and multivesicular bodies (MVBs), which form intraluminal vesicles (ILVs) carrying nucleic acids and proteins. ILVs are released as exosomes upon MVB–plasma membrane fusion. Ectosomes (100 nm–1 μm) bud directly from the plasma membrane. Apoptotic bodies (>1 μm) are formed during programmed cell disintegration. EVs facilitate intercellular communication via transport of bioactive molecules. Created in BioRender. Yang, Zhuoyue. (2026) https://BioRender.com/oamkakl (accessed on 4 March 2026).

EVs are secreted by almost all cell types and are widely distributed in various bodily fluids, including blood, urine, saliva, milk, and bile [31]. Based on the differences in physical characteristics and biological pathways, EVs are mainly classified into three categories (Figure 3): exosomes (30–150 nm), microvesicles or ectosomes (100 nm–1 μm) and apoptotic bodies (>1 μm) [32,33]. The generation of exosomes begins with the formation of early endosomes by plasma membrane invagination, which gradually mature into late endosomes, subsequently forming multivesicular bodies (MVBs). Intracavitary vesicles (ILVs) inside the MVBs incorporate proteins and mRNAs during the maturation of early endosomes. Ultimately, ILVs are released by exocytosis upon the fusion of MVBs with the plasma membrane, known as the final form of exosomes [34]. Structurally, exosomes resemble liposomes as they both contain diverse biomolecules, but exosomes exhibit greater compositional complexities. At present, nearly 100,000 proteins and more than 1000 lipids have been identified in exosomes. Protein components in exosomes include heat shock proteins (HSP60, HSP70), membrane transport and fusion proteins, as well as tetraspanins (CD9, CD63 and CD81), etc. [35].

In contrast, the formation of ectosomes depends heavily on dynamic plasma membrane restructuring. These vesicles with a size range of 100 nm to 1 μm in diameter bud directly from the membrane into the extracellular space. Apoptotic bodies are released at the programmed stage of cell membrane shrinkage and invagination, which contain DNA and organelles to form vesicles [30]. EVs interact dynamically with target cells through ligand–receptor interaction, membrane fusion as well as endocytosis, and they carry biologically active molecules, including proteins, lipids, nucleic acids, and metabolites, that play key roles in intercellular communication [36,37]. In addition, PDEVs are generated through unique biogenesis pathways that reflect their distinct cellular architecture. One such pathway involves the exocyst-positive organelle (EXPO), a double-membraned structure that fuses with the plasma membrane to release vesicles extracellularly. Another important route is the vacuolar pathway, wherein MVBs may deliver intraluminal vesicles (ILVs) to vacuoles, which subsequently fuse with the plasma membrane to release the remaining ILVs outside the cell [38,39,40].

### 3.2. Isolation of Natural Biological Resources Derived EVs

As mentioned above, natural biological-resource-derived EVs, including Ti-EVs in mammals and PDEVs are advantageous over C-EVs with regard to higher extraction efficiency and greater physiological relevance [41]. Furthermore, growing evidence have indicated that Ti-EVs harbor more active components than C-EVs, attributable to their histological background and tissue specificity [42,43]. Meanwhile, multiple novel biomarkers for diverse diseases have been elucidated by utilizing Ti-EVs. PDEVs that are present in various tissues of plants have been successfully isolated from over 20 edible plant species [43], and they are not only involved in key physiological processes in plants, such as anti-pathogen defense and stress response, but also have the potential to cross-border regulate human cellular functions [44]. Despite the structural similarities with mammalian exosomes, PDEVs have the unique advantages of excellent stability, low immunogenicity, inherent antioxidant components, and cost-effectiveness [45]. Owing to their natural origin and distinctive biophysical properties, PDEVs hold promising application prospects as both therapeutic agents and drug delivery carriers [46]. Obtaining high-purity EVs from mammalian tissues and plants is an essential prerequisite for studying their biological applications. Both mammalian tissues and plants need to undergo sample pre-preparation prior to isolation to obtain purified EVs. In this section, we comprehensively summarize and discuss the sample pre-preparation protocols and isolation methodologies for these natural EV sources.

#### 3.2.1. Sample Pre-Preparation of Natural Biological Resources for Extracting EVs

**Figure 4:**
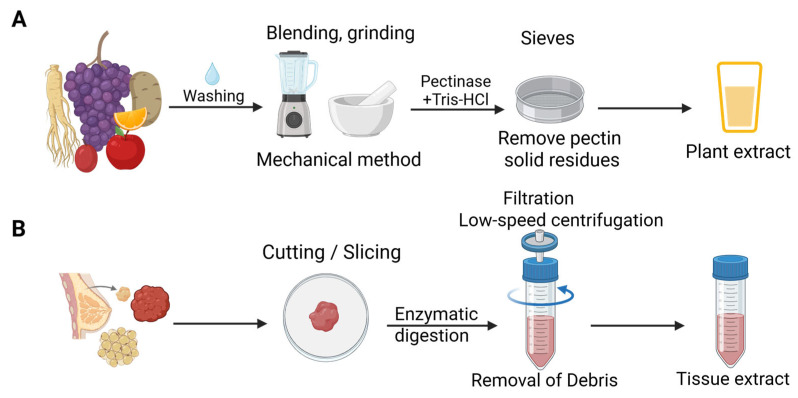
Sample pre-preparation of natural biological resources for extracting EVs. (**A**) PDEVs; (**B**) Ti-EVs. Created in BioRender. Yang, Zhuoyue. (2026) https://BioRender.com/p0je0y7 (accessed on 4 March 2026).

Although the isolation protocols for Ti-EVs and PDEVs are commonly created based on that for C-EVs, both of them require an extra sample pre-preparation process after dissociating mammalian tissues and plants without destroying cells, which is well believed to be extremely important for improving the quality and yield of EVs (Figure 4) [42,43,46]. The specific details of sample pre-preparation vary considerably depending on the type of tissue and plant, but the general pretreatment process remains similar. For plants, the surface of the sample is washed with distilled water, followed by being crushed through gentle mechanical methods (Table 1), such as extrusion and grinding using a commercial juicer and stirrer [47,48]. However, it should be noted that cell debris, cytoplasmic proteins, and other ECM components might coexist with EVs during the process, increasing the likelihood of EV contamination [49]. In addition, in order to improve the purity of EVs, Tris-HCl, pectinase and sieves are typically applied in the pretreatment stage to remove pectin and solid residues, respectively [50]. The high purity of PDEVs cannot be guaranteed due to the pretreatment process, and PDEVs usually suffer from unique contaminants including polysaccharides, polyphenols, chloroplasts, and highly abundant proteins, which change vesicle structure and hinder cellular uptake. More importantly, these contaminants could severely impair PDEVs’ stability and biological function, thus restricting their clinical translation. Therefore, the establishment of efficient and mild purification systems as well as standardized quality control processes is crucial to advance the therapeutic application of PDEVs [51].

To obtain Ti-EVs that are entrapped in tissue interstitium, mammalian tissues are usually subjected to enzymatic digestion in conjunction with mechanical disruption prior to EV isolation [52,53]. Fresh tissue samples were gently cut or sliced into small fragments, thereby reducing the risk of EV contamination by intracellular vesicles. Subsequently, the ECM is degraded by enzymatic digestion to dissociate cells and release entrapped EVs, such as collagenases and pepsin [54,55]. There exists apparent variability in EV quality and yield due to the different tissue sources; thus, digestion conditions such as enzyme type, concentration, time, and temperature require optimization according to the tissue type to achieve optimal digestion efficiency. After digestion, residual tissue fragments and large cellular debris are typically removed by filtration or low-speed centrifugation to obtain preliminary lysate-containing EVs [55].

**Table 1:** Pre-preparation and isolation methods of plant-derived extracellular vesicles.

Source	Part Used	Sample State	Mean Particle Size	Centrifugation Speed	Pre-Preparation	Isolation Method	Ref
Ginseng	Root	Fresh	142 nm	/	Grinding machine	Ultracentrifugation	[56]
Potato	Whole	Fresh	60 nm	120,000 g	Grinding machine	Ultracentrifugation	[57]
*Olea europaea*	Leaves	Fresh	140 nm	10,000 g	Grinding machine	SEC	[58]
Ginseng	Root	Fresh	92.04 nm	150,000 g	Grinding machine	Ultracentrifugation, DGC	[59]
*Aloe saponaria*	Peel	Fresh	<200 nm	10,000 g	Grinding machine	PEG precipitation	[60]
Pomegranate	Arils	Fresh	148.7 nm	20,000 g	Grinding machine	TFF; SEC	[61]
*Dendrobium*	Whole	Fresh	81.8 nm	150,000 g	Grinding machine; Tris-HCL	Sucrose DGC	[62]
Wheat	Grass juice	Fresh	40–100 nm	16,000 g	Grinding machine	Ultracentrifugation	[63]
Grapefruit	Whole	Fresh	132 nm	1000 g	Grinding machine	Aqueous two-phase system	[64]

SEC, size-exclusion chromatography; PEG, polyethylene glycol; DGC, density gradient centrifugation; TFF, tangential flow filtration.

#### 3.2.2. Separation of Natural Biological-Resource-Derived EVs

**Figure 5:**
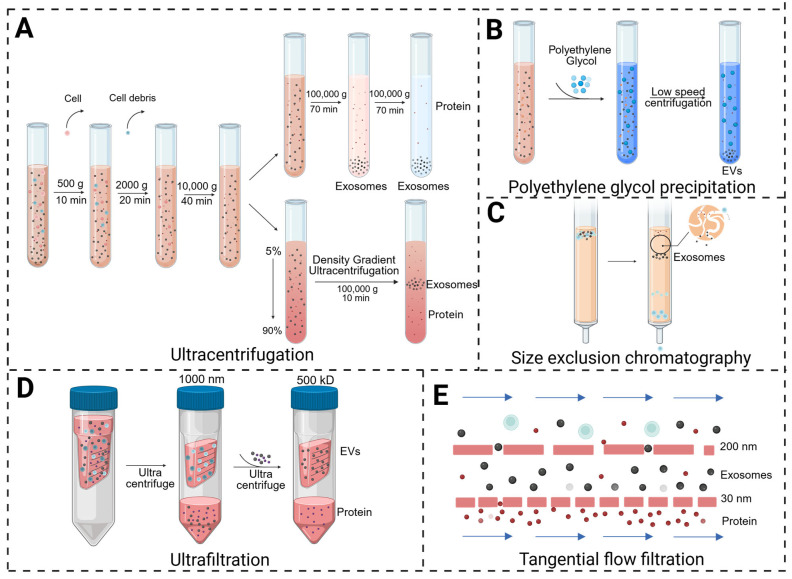
Common techniques for EV isolation. (**A**) Ultracentrifugation (differential and density gradient); (**B**) polyethylene glycol precipitation; (**C**) size-exclusion chromatography; (**D**) ultrafiltration; (**E**) tangential flow filtration. Each technique exploits distinct physicochemical properties of EVs for separation. Created in BioRender. Yang, Zhuoyue. (2026) https://BioRender.com/qkqxx74 (accessed on 4 March 2026).

Researchers have developed a variety of separation and purification methods based on the physical and chemical properties of EVs. Herein, several commonly reported separation methods of EVs are compared and schematically illustrated in Figure 5 [65]. Ultracentrifugation is the most broadly adopted approach for EV isolation, including differential ultracentrifugation and density gradient ultracentrifugation [66]. Differential ultracentrifugation separates particles primarily based on differences in size, density and shape. According to the difference in size and density between exosomes and other components in the sample, the non-exosome components were gradually removed through several rounds of centrifugation with increasing speeds, after which the exosomes were precipitated and re-suspended. Despite differential ultracentrifugation being relatively well-established and suitable for the isolation of most EV types, it still has some disadvantages, including being time-consuming and requiring an expensive centrifuge, as well as possible damage to the integrity of exosomes. Density gradient centrifugation employs media with continuous density gradients to make particles of different densities, such as exosomes and protein aggregates, settle in the corresponding layer according to their specific density during centrifugation [67]. The exosomes obtained by density gradient centrifugation have better integrity, biological activity and purity. In comparison, traditional differential ultracentrifugation relies on the difference in the sedimentation velocities of the particles, and it is difficult to completely distinguish exosomes from other vesicles and impurities.

Beyond ultracentrifugation, several alternative strategies have been developed for effective EV isolation. Polymer-based precipitation is also widely used in the purification of exosomes, which mainly employs polyethylene glycol (PEG) and other polymers to competitively bind water molecules, forcing exosomes to precipitate from the solution. This method is easy to operate, but nucleic acids and proteins are inevitably simultaneously precipitated during the separation process, leading to the fact that a complex post-processing process is generally required [68]. Ultrafiltration is a size-based separation method, which requires the selection of cut-off membranes with different pore sizes. Ultrafiltration is usually performed in conjunction with centrifugation, which allows the samples to pass through the filter membrane so that nanoparticles of different sizes and dimensions are separated. However, the irreversible accumulation of macromolecules on the ultrafiltration membrane greatly compromises the efficiency of ultrafiltration. In addition, some nanoparticles close to the size of the exosome particles may also be retained, lowering EV purity. There exists an advanced form of ultrafiltration called tangential flow filtration (TFF), which is distinct from traditional dead-end filtration. TFF relies on shear force to prevent membrane pore blockage and utilizes transmembrane pressure to drive small molecules through the membrane, further improving separation efficiency [69]. Size-exclusion chromatography (SEC) is also a method for separating particles according to their sizes, applying an SEC column containing porous spherical beads filled with resin. When the solution with exosomes flows through the SEC column, smaller particles are captured in the pores, whereas larger particles are eluted. SEC has been successfully utilized for the separation of EVs from a great number of biological samples and can be used either alone or in combination with other separation methods [70].

Different isolation methods exhibit distinct performances in terms of purity, yield, scalability, and compatibility with clinical manufacturing. Differential ultracentrifugation and density gradient ultracentrifugation provide high purities and are widely recognized as the gold standard but show moderate yield, low scalability, and limited suitability for large-scale clinical production. PEG-based polymer precipitation achieves high yield and excellent scalability, yet suffers from low to moderate purity and poor clinical compatibility due to co-precipitated impurities and potential polymer residues. Ultrafiltration offers moderate to high yields and scalabilities with moderate purity, serving as a practical concentration step in clinical workflows but requiring further purification. The SEC delivers high purity with minimal vesicle damage, moderate yield, and good scalability, representing a clinically favorable approach.

The choice of isolation method profoundly impacts the yield, purity, and functionality of the resulting EVs, which is a critical consideration for translational research. Even if the various isolation methods mentioned above, such as density gradient ultracentrifugation, ultrafiltration, polymer-based precipitation, and SEC, have been frequently applied to the separation of Ti-EVs and PDEVs [71,72,73], ultracentrifugation is still the preferred method for most studies on natural biological-resource-derived EVs. Researchers usually centrifuge plant samples at low speeds between 500 and 10,000 g to remove cells and plant debris, and then the supernatant is subjected to centrifugation at a high speed of >100,000 g for more than 1 h to recover PDEVs. Notably, it is difficult to effectively distinguish Ti-EV subgroups by differential centrifugation, which is also the most widely applied method for Ti-EV isolation [42]. According to particle size, Ti-EVs are usually divided into large EVs and small EVs that mainly contain microvesicles and exosomes respectively, and their precise separation and purification remains to be an obstacle.

## 4. Skin Repair Activity of Natural Biological-Resource-Derived EVs

### 4.1. Therapeutic Effects of EVs on Wound Healing

Wound healing represents a complex pathophysiological process dynamically regulated by multiple stages and involving diverse cell types [74]. Based on distinct healing characteristics, clinical wounds are broadly categorized into acute and chronic wounds. Acute wounds can readily achieve self-healing through a cascade of repair mechanisms under physiological conditions, which is highly difficult for chronic wounds due to their complex pathological mechanisms, long healing cycles (typically exceeding 12 weeks) and poor prognosis. The unfeasible restoration of chronic wounds not only significantly diminishes the life quality of patients but is also accompanied by a high risk of death and serious cosmetic problems [75]. Therefore, it is of great clinical value to explore the accelerated mechanism of chronic wound healing and develop corresponding therapeutic solutions [76,77,78]. In this section, the beneficial effects of mammalian Ti-EVs and PDEVs on wound healing as well as the underlying cellular and molecular bases are summarized in detail (Table 2).

#### 4.1.1. Inflammatory Modulation by EVs in Wound Healing

The inflammatory phase is well believed to be the cornerstone of the wound healing process. At this stage, neutrophils arrive at inflammatory sites followed by monocytes that are rapidly differentiated into macrophages, which are responsible for removing remanent cell debris and excessive neutrophils. In short, the core roles of the inflammatory phase are to clean up pathogens and necrotic tissue, initiate repair signals, as well as prevent infection. The normal wound healing process heavily benefits from a well-controlled inflammatory response, whereas the formation of chronic wounds is closely related to the loss of fine control over the inflammatory response. During chronic wounds, excessive inflammation reactions seriously destroy the dynamic balance of the healing process, resulting in wound stagnation during the inflammatory period and final failure to enter the repair stage [79]. Therefore, the precise regulation of inflammatory response can tremendously drive the development of normal wound healing.

Accumulating evidence have indicated that Ti-EVs exert significant anti-inflammatory effects. For instance, Pan et al. used three different methods, including mechanical, collagenase–digestive, and constant in vitro collective methods, to prepare therapy-grade EVs from adipose tissue and explore their respective activities. The results demonstrated that adipose-tissue-derived EVs (AT-EVs) significantly decreased the positive rates of M1 macrophage markers in vitro, including inducible nitric oxide synthase (iNOS) and cluster of differentiation 86 (CD86), concurrently reducing the expression of pro-inflammatory cytokines such as interleukin-6 (IL-6) and tumor necrosis factor-α (TNF-α) [80]. Further diabetic wound healing studies in vivo showed that the inflammatory marker CD68 was significantly reduced upon AT-EV treatment, while the proportion of M2 macrophages simultaneously increased [80]. Consistent with this immunomodulatory effect, Dong et al. found that AT-EVs downregulated M1-associated genes (IL-6, TNF-α), while upregulating M2-associated markers, including arginase-1 (ARG1), transforming growth factor-β1 (TGF-β1), interleukin-10 (IL-10) and CD206. Moreover, culturing M1 macrophages with 100 μg/mL of AT-EVs promoted the phenotypic shift from M1 to M2 [81].

Plants have been recognized to be efficacious in the treatment of skin wounds for a long period. IL-6 and IL-1β have strong pro-inflammatory activities and can induce a variety of pro-inflammatory mediators. It has been reported that *aloe saponaria*-derived EVs (AS-EVs) inhibited the expression of IL-6 and IL-1β in RAW 264.7 cells induced by lipopolysaccharide (LPS) [60]. In addition, *dendrobium*-derived nanovesicle (DDNV) treatment effectively inhibited the expression of IL-1β and accelerated tissue repair in mouse wound skin [62]. Ginseng-derived nanoparticles (GDNPs) significantly reduced the expression of inflammatory factors iNOS and NF-кB, effectively improving inflammatory stimulation [82]. Studies have also shown that EVs from pomegranate juice (PgEVs) significantly inhibited LPS-induced NF-кB production [61]. Similarly, red onion-derived EVs (RDNVs) demonstrated significant anti-inflammatory effects by polarizing M1 macrophages into the M2 phenotype and substantially reducing the expression of pro-inflammatory genes. Histological analysis confirmed a significant reduction in inflammatory cell infiltration in RDNV-treated wounds [83]. Furthermore, coriander-derived exosome-like nanovesicles (CDENs) are isolated and characterized. CDENs-based hydrogel effectively relieved inflammation by facilitating macrophage M2 polarization during the inflammation phase. Mechanistically, it activated the nuclear factor erythroid 2-related factor 2 (Nrf2) signaling pathway, which enhanced the antioxidant enzyme defense system and reduced the inflammatory response [84]. *Paris polyphylla* var. *yunnanensi-*derived EVs (PPEVs) were demonstrated to have significant anti-inflammatory properties by reducing the expression of pro-inflammatory cytokines (IL-1β, IL-6, and TNF-α) by more than 1.5 times. Similarly, EVs derived from *Crithmum maritimum* callus (Cm-callus EVs) at concentrations of 2.6 × 10^8^ to 2.6 × 10^9^ particles/mL upregulated TGF-β1 mRNA expression by 1.4 to 2.0 fold in human dermal fibroblasts, suggesting a potential anti-inflammatory regulatory effect [85]. Additionally, they exhibited a strong ROS scavenging activity, with a gradual decrease in fluorescence intensity as their concentration increased, reducing ROS levels by 50% at 20 μg/mL [86].

#### 4.1.2. EVs Promote Angiogenesis in Wound Healing

Angiogenesis plays a pivotal role in the proliferative phase of wound healing, which provides oxygen, nutrition and metabolic support for tissue repair by forming a new vascular network. Granulation tissue deposition requires blood vessels to supply nutrition, and vascular dysfunction may lead to the formation of chronic wounds [87]. Therefore, angiogenesis constitutes a critical determinant of wound healing efficacy, and its spatiotemporal regulation directly governs tissue repair outcomes.

AT-EVs have been evidenced to potentially promote the migration and neovascularization of human umbilical vein endothelial cells (HUVECs) [60,73]. When endothelial cells are cultured with AT-EVs, the mRNA expression of angiogenesis markers are upregulated, such as platelet endothelial cell adhesion molecule-1 (CD31), VEGF, fibroblast growth factor 2 (FGF2) and angiogenin, which are able to promote the formation of tubular structures in endothelial cells [81]. In the mouse wound model, AT-EVs can significantly enhance the expression of CD31 and alpha-smooth muscle actin (α-SMA), suggesting their angiogenic activity in diabetic wounds [88]. Besides AT-EVs, the role of PDEVs in angiogenesis during wound healing has also been extensively studied. For example, wheat exosomes were revealed to have the ability to increase HUVEC migration and tube-like structure formation, suggesting a functional link between wheat exosome and angiogenesis [63]. Similarly, grapefruit-derived EVs were also demonstrated to display favorable effects on the angiogenesis capacities of HUVECs in the tube formation assay [64]. In addition, Yang et al. found that ginseng-derived nanoparticles significantly increased tube-like structure formation by promoting the development of branches, thereby accelerating vascularization during wound healing [82].

#### 4.1.3. EVs Promote Cell Proliferation and ECM Remodeling in Wound Healing

Fibroblasts are the main effector cells responsible for skin wound healing; thus, their proliferation and migration are extremely pivotal. Studies have shown that AT-EVs can facilitate the proliferation and migration of epidermal cells (HaCaT) and human dermal fibroblasts (HDF) and upregulate epidermal formation marker cytokeratin 14 (CK14) and cell proliferation marker Ki67, finally promoting diabetic wound healing by re-epithelialization with collagen deposition and rearrangement [80]. Moreover, white-adipose-tissue-derived EVs (WAT-EVs) showed more stable wound repair activities than brown-adipose-tissue-derived EVs (BAT-EVs) [88]. Mechanically, exosome miRNAs in Ti-EVs critically regulate multiple wound healing processes, including cell proliferation and ECM remodeling. For instance, miR-92a derived from AT-EVs activates the large tumor suppressor kinase 2 (LATS2) and Yes-associated protein (YAP)/Transcriptional coactivator with PDZ-binding motif (TAZ) signaling axis, thereby enhancing fibroblast proliferation as well as the production of collagen and fibronectin [89]. In addition, researchers have also obtained vesicles from human cancer tissues and rat skin tissues, elucidating their effects on wound healing. The results showed that EVs derived from breast cancer tissues and oral squamous cell carcinoma tissues had the potential to accelerate cell proliferation [90,91]. Rat-skin-tissue-derived vesicles can not only promote the proliferation and migration of HaCaT but also facilitate granulation tissue formation and ECM deposition, thereby increasing the wound closure rate of mice [11].

PDEVs are reportedly potential facilitators for cell proliferation and ECM remodeling during wound healing. The study found that none of the wheat-derived vesicles at 200 μg/mL exhibited cytotoxic effects and that wheat-derived vesicles enhanced the proliferation of HaCaT and HDF cells in a dose-dependent manner, while also increasing collagen expression in HDFs, thereby serving as an alternative to fibrin clots to further promote wound healing [63]. Grapefruit-derived EV treatment increased the expression of laminin, fibronectin, vimentin and epidermal growth factor in a dose-dependent manner, thereby promoting ECM remodeling [64]. The mouse wound experiment showed that *dendrobium*-derived nanovesicles induced the activation of the interleukin-17 (IL-17) signaling pathway and indirectly regulated the NF-κB signaling pathway, NETosis and the TNF signaling pathway to modulate apoptosis as well as cell senescence, ultimately promoting cell proliferation [62]. Furthermore, GDNPs dose-dependently enhanced the proliferation and migration of HaCaT and foreskin fibroblasts through extracellular signal-regulated kinase (ERK) and protein kinase B (AKT)/mammalian target of rapamycin (mTOR) pathway activation, while upregulating the expression of ECM remodeling mediators, such as MMP-1, fibronectin-1, elastin-1 and collagen I [82]. Additionally, exosome-like vesicles derived from LE callus (LELVs) can promote fibroblast proliferation in a dose-dependent manner after 48 h of treatment, with the optimal pro-proliferative effect observed at a concentration of 1 × 10^10^ particles/mL. They also accelerated re-epithelialization, increased collagen deposition, and enhanced ECM remodeling in mouse wounds [92]. Likewise, Cm-callus EVs at concentrations of 2.6 × 10^8^ to 2.6 × 10^9^ particles/mL dose-dependently accelerated wound closure by up to 70% [85]. Treatment with RDNVs enhanced wound healing processes by improving granulation tissue formation and accelerating re-epithelialization [83]. The CDENs were found to promote HaCaT cell migration. Furthermore, during the remodeling phase of wound healing, the CDENs-hydrogel expedited collagen deposition [84]. PPEVs promoted expression of the epidermal growth factor and collagen mRNA, indicating enhanced tissue regeneration [86].

While Ti-EVs show promising therapeutic potential and no toxicity in most studies [93,94], their clinical translation is accompanied by unique challenges. First, the biological activity and composition of Ti-EVs can be influenced by donor-specific factors such as age, health status, and lifestyle. For instance, Lou et al. found that skin-tissue-derived EVs could promote wound healing, and EVs from neonatal skin had a higher repair potency than those from adult skin [11]. Second, the use of EVs derived from pathological tissues, such as tumors, might have potentially undesirable effects and require extreme caution. Although studies have reported the pro-healing effects of tumor-derived EVs, they may also carry oncogenic proteins, miRNAs, and immunosuppressive factors, with potential risks of promoting tumor progression or metastasis. Accumulating evidence highlights that EVs are robust communicators for cell-to-cell crosstalk in the tumor microenvironment. Indeed, tumor-derived EVs extensively participate in the complex processes of cancer occurrence and progression via abundant bioactive substances inherited from parental tumor cells [95]. Therefore, their application should currently be restricted to mechanistic research, and stringent safety profiling is imperative before any therapeutic consideration.

**Table 2:** The activities of natural-resource-derived EVs in promoting wound healing.

EV Source	Key Mechanism	Centrifugation Speed	Source Details	Delivery Method	Target Cells	Mean Particle Size	Study Type	Potential Application	Ref
AT-EVs	Promote angiogenesis and fibroblast/keratinocyte proliferation; Inhibit M1-to-M2 polarization	10,000× *g*	Abdomen	PBS	Fibroblasts, keratinocytes	25–240 nm	in vitro + in vivo	Diabetic wound healing	[81]
WAT-EVs	Increase cell survival rate; Promote angiogenesis; Inhibit oxidative stress	140,000× *g*	Inguinal and interscapular	PBS	HUVEC, fibroblasts, HaCaT	134.5 nm	in vitro + in vivo	Diabetic wound healing	[88]
Breast carcinoma-EVs	Promote cell proliferation	150,000× *g*	Breast tumor tissue	PBS	Fibroblasts	110.3 nm	in vitro	Diabetic wound healing	[90]
GDNPs	Regulate ERK/AKT/mTOR pathway; Promote skin cell proliferation; Upregulate MMP-1 and collagen	150,000× *g*	/; Whole;	/	HaCaT, Fibroblasts, HUVEC	215.2 nm	in vitro + in vivo	Wound healing	[82]
DDNVs	Regulate IL-1β/IL-17 pathway; Inhibit NF-KB pathway; Inhibit NETosis pathway	150,000× *g*	Fresh; Whole	/	Inflammatory cells, HUVEC	81.8 nm	in vitro + in vivo	Diabetic wound healing	[62]
Wheat-EVs	Promote cell proliferation; Increase collagen expression; Inhibit apoptosis	16,000× *g*	Fresh; Grass juice	PBS	HUVEC, fibroblasts, HaCaT	40–100 nm	in vitro	Chronic wound healing	[63]
RDNVs	Induce M2 phenotype; Inhibit pro-inflammatory genes; Accelerate tissue regeneration	16,000× *g*	Fresh; Peels	PBS	Macrophages, fibroblasts	397.5 nm	in vitro + in vivo	Wound healing	[83]
CDENs	Facilitate M2 polarization; Activate Nrf2 pathway; Promote cell migration; Expedite collagen deposition	/	/	/	Macrophages, HaCaT, fibroblasts	/	/	Wound healing	[84]
PPEVs	Reduce pro-inflammatory cytokines (IL-1β, IL-6, TNF-α); Scavenge ROS; Promote EGF and collagen mRNA expression	15,000× *g*	Fresh; Rhizomes	Hydrogel	Immune cells, Fibroblasts	156.8 nm	in vitro + in vivo	Wound healing	[86]
LELVs	Promote cell proliferation and collagen synthesis; Accelerate re-epithelialization	3500× *g*	Fresh; Callus	/	Fibroblasts	129 nm	in vitro + in vivo	Wound healing	[92]
Cm-callus EVs	Promote migration and wound healing in fibroblasts	150,000× *g*	Fresh; Leaves callus	/	Fibroblasts	136.6 nm	in vitro	Wound healing	[85]

HUVEC, human umbilical vein endothelial cells; EGF, epidermal growth factor; NETosis, neutrophil extracellular trap formation; IL-1β, interleukin-1 beta; IL-17, interleukin-17; NF-KB, nuclear factor kappa-light-chain-enhancer of activated B cells; TNF-α, tumor necrosis factor alpha; ROS, reactive oxygen species; Nrf2, nuclear factor erythroid 2-related factor 2; ERK, extracellular signal-regulated kinases; AKT, protein kinase B; mTOR, mammalian target of rapamycin; MMP-1, matrix metalloproteinase-1.

### 4.2. Therapeutic and Reparative Effects of EVs on Skin Photoaging

#### 4.2.1. EVs Attenuate Oxidative Stress During Anti-Photoaging

UVA penetrates into the dermis and indirectly produces ROS through a photosensitive reaction. UVB acts on the epidermis, directly damaging DNA and generating ROS. Oxidative stress leads to structural lesion and functional decline in skin through ROS-mediated molecular damage, dysregulated signaling pathways, and inflammatory cascades, probably leading to sunburn, premature aging and carcinogenic effects. Growing studies have shown that exosomes can effectively alleviate oxidative stress in the body (Table 3). For example, nanovesicles derived from *olea europaea* leaf (OLELNVs) have been shown to contain superoxide dismutase (SOD), which is an important antioxidant enzyme and plays a key role in the antioxidant defense system. OLELNVs reduced ROS levels and restored SOD activity in a dose-dependent manner and showed a stronger free radical scavenging ability than *olea europaea* leaf extract [58]. Choi et al. found that ginseng-root-derived exosomes (GrEVs) reduced the level of ROS in HaCaT cells in a dose-dependent manner, with the ROS level reaching its minimum at a concentration of 2 × 10^9^ particles/mL [56]. Similarly, exosomes derived from *Iris* rhizomes (Iris-exos) have also been shown to reduce ROS levels in a dose-dependent manner. After Iris-exos treatment, the expression of heme oxygenase-1 (HO-1), catalase, glutathione synthetase (GSS), glutathione peroxidase 1 (GPx1), and SOD in epidermal keratinocytes was upregulated, which further verified the antioxidant potential of Iris-exos [96]. The potato-derived EVs not only showed a concentration-dependent 1,1-diphenyl-2-picryl-hydrazyl radical (DPPH) scavenging activity but also further increased the expression level of cytoprotective glutathione s-transferase alpha 4 (GSTA4) [57]. GSTA4 belongs to α-type cytosolic soluble GST, which can be used as a phase II detoxification enzyme for antioxidant damage. GSTA4 is adaptive to oxidative stress and provides protection against oxidative stress for cells [97]. Setiadi et al. have formulated and developed a kind of gel, and combined it with exosomes derived from cherries. The results showed that a formulation incorporating 0.25% golden cherry exosomes had the best stability compared with other concentrations. The incorporated exosomes showed antioxidant activity, thus reducing the risk of photoaging [98]. Furthermore, *aloe*-derived exosome-like nanoparticles (ADNPs) promoted the nuclear translocation of Nrf2 and alleviated oxidative stress induced by UV exposure [99]. In vivo, ADNPs reduced malondialdehyde (MDA) and SOD levels in mouse skin tissue. Similarly, grape-derived exosome-like nanoparticles (GENs) were shown to reduce ROS levels in UVB-radiated HaCaT. Additionally, in the mouse photoaging model, GEN treatment increased the activity of SOD and decreased MDA levels [100]. Mechanistically, EVs isolated from *Ecklonia cava* (EC-EVs) significantly downregulated NADPH oxidase isoforms (NOX1, NOX2, NOX4) and oxidative DNA damage marker 8-hydroxy-2′-deoxyguanosine (8-OHdG) in senescent keratinocytes, thereby alleviating cellular oxidative stress. In order to further elucidate the effect of EC-EVs on aging skin, EC-EVs were injected into mice by Batsukh et al. The results showed that they not only drastically downregulated the expression of NOX1, NOX2, NOX4 but also significantly reduced 8-OHdG expression levels in aging skin, suggesting that EC-EV treatment could successfully reduce oxidative stress [101].

#### 4.2.2. EVs Inhibit Inflammatory Response During Anti-Photoaging

UV-induced inflammation plays a critical role in the process of photoaging [102]. Long-term UV exposure leads to a continuous low-grade inflammatory state of the skin and causes skin cells to produce a large number of inflammatory factors, especially IL-6, which triggers local and systemic inflammatory responses. GrEVs and OLELNVs can improve inflammation and immune responses by inhibiting the secretion of pro-inflammatory cytokine IL-6 (Table 3). Studies have shown that GrEV treatment can also reduce the expression of cyclooxygenase-2, which is the dominant regulator of UV-induced skin inflammation, photoaging and carcinogenesis [56]. In addition, OLELNV treatment significantly downregulated the NF-κB signaling cascade, which was a key mediator of inflammatory response. This suggests that GrEVs and OLELNVs may help to restore UV-induced inflammation to a certain extent [58,59]. Another study evidenced that potato-derived EVs can not only downregulate the expression of IL-6 in a dose-dependent manner but also inhibit the expression of TNF-α [57]. *Lavender* exosome-like nanoparticles (LELNs) exhibited similar activities to potato-derived EVs. Key miRNAs in LELNs, particularly cpa-miR166e and zma-miR166h-3p from the miR166 family, played a critical role in regulating inflammation [103]. In a similar manner, GEN treatment downregulated the mRNA expressions of IL-6 and IL-1β. Single-cell RNA sequencing further supported the anti-inflammatory role of GENs by revealing restored cellular communication and reduced inflammation-related signaling pathways in photoaged skin [100].

#### 4.2.3. EVs Mitigate DNA Damage and Cell Senescence

UVA causes oxidative DNA damage by producing ROS, which leads to the consumption of cellular antioxidants and antioxidant enzymes (SOD, catalase), and initiates DNA damage [104]. After UVB is absorbed by DNA, it induces the formation of thymidine dimer that can hinder DNA replication and transcription [105]. DNA alternation is the most critical, and the accumulation of DNA damage can lead to cell senescence and apoptosis.

ADNPs alleviated DNA damage induced by UV exposure and inhibited the elevation of senescence-associated β-galactosidase (SA-β-gal) and senescence-associated secretory phenotype (SASP), thereby mitigating cell senescence [99]. GrEV treatment significantly reduced SA-β-gal activity and the melanin level of senescent melanocytes in a dose-dependent manner, as well as improved the photoaging phenotype. In addition, the mRNA expression levels of other senescence-associated markers were also gradually downregulated with the increase in GrEV concentration, further demonstrating that GrEVs effectively inhibited cell senescence [59]. Another study by Choi et al. showed that GrEVs reduced the mRNA expression level of pro-apoptotic molecules, including Bcl-2-associated X protein (BAX) and caspase-1, -3, -6, -7, -8, which were significantly increased under UV radiation, further evidencing that GrEVs could effectively alleviate cell senescence [56]. It has been reported that Iris-EV treatment significantly reduced the number of SA-β-gal-positive cells in a dose-dependent manner and dramatically downregulated the expression of p21, which is a cell cycle-dependent kinase inhibitor and an important marker of aging. In addition, Iris-EVs may also downregulate the expression of p53 and p21 by reducing JNK and p38-mediated p53-p21 signaling, thereby effectively inhibiting cell senescence [96].

Similarly, GEN treatment reduced SA-β-gal activity in senescent HaCaT and downregulated p53 as well as p21. Single-cell sequencing identified that GENs restored the population of epithelial subpopulation C10, which is highly susceptible to UV-induced senescence [100]. EC-EVs could significantly reduce the expression of senescence markers P21 and P16 in keratinocytes. Moreover, EC-EV treatment improved the aging-related changes in keratinocytes, upregulating the expression of HSP70 and downregulating the expression of TNF-α, MAPK, NF-κB, AP-1 and MMP. Similar results were also found in aged mouse experiments [101]. In another study, EVs derived from adipose tissue were injected into the face of patients for clinical research. After evaluation by the Berardesca scale, numerical rating scale and modified Vancouver scale, the results showed that EV treatment induced decreased signs of tissue aging in all patients. In addition, the study indicated that it was possible to use a simple ultrafiltration system to extract signal microbubbles and that the ultrafiltrates could be effectively used for therapeutic treatment [106].

#### 4.2.4. EVs Promote ECM Synthesis During Photoaging

Collagen and elastin that are responsible for supporting skin structure in the dermis, playing vital roles in skin growth and elasticity. When exposed to UV light, MMP is activated, which leads to the excessive degradation of collagen and elastin, resulting in aging symptoms such as wrinkles and decreased skin elasticity. It has been reported that exosome-like nanovesicles isolated from callus induced from apple fruit can promote the expression of collagen type I alpha 1 chain (COL1A1) and fibrillin-1 (FBN1) in UVA-radiated HDF and further promote collagen synthesis in HDF, thereby improving the skin barrier and anti-aging [107]. FBN1 is the microfiber core that constitutes elastic fibers, while COL1A1 constitutes type I collagen fibers, both providing mechanical support and forming a dense fiber network in the ECM. The studies by Lee et al. showed that potato-derived exosomes downregulated the expression of MMP1, 2 and 9 while regulating the expression of MMP1 in a concentration-dependent manner and preventing collagen degradation [57]. Similarly, LELNs improved collagen preservation in vivo, with the optimal effect observed at a concentration of 1 μg/μL. Functional enrichment analysis revealed that miRNAs in LLENs were involved in collagen synthesis pathways and played key roles in regulating collagen metabolism [103]. Furthermore, Masson staining showed that GENs increased collagen deposition in the dermis of photoaged mice. GENs also downregulated MMP1 expression, which was responsible for collagen degradation [100]. Cm-callus EVs dose-dependently upregulated the mRNA expressions of COL1A1, collagen type I alpha 2 chain (COL1A2), vascular endothelial growth factor A (VEGFA), and TGF-β1 and suppressed MMP1 expression in human dermal fibroblasts. In a UVA-induced photodamage model, treatment with 10% Cm-callus EVs at a concentration of 2.6 × 10^9^ particles/mL restored COL1A1 protein expression to 92% of that observed in non-irradiated controls [85].

**Table 3:** The activities of EVs in promoting photodamage repair.

EVs Source	Key Mechanism	Centrifugation Speed	Source Details	Delivery Method	Target Cells	Mean Particle Size	Study Type	Potential Activities	Ref
OLELNVs	Reduce ROS; Restore SOD activity; Inhibit NF-κB pathway	10,000× *g*	Fresh; Leaves	Hydrogel	HDF, HaCaT	140 nm	in vitro + in vivo	Anti-photoaging; Anti-inflammatory	[58]
GrEVs	Reduce ROS/SA-β-gal; Inhibit IL-6/apoptotic factors	/	Fresh; Root	/	HaCaT	142 nm	in vitro	Anti-photoaging	[56]
Iris-exos	Activate antioxidant enzymes; Inhibit p21 pathway; Decrease SA-β-gal activity	10,000× *g*	Dry; Rhizomes	/	Human epidermal keratinocytes	172 nm	in vitro	Antioxidant; Anti-photoaging	[96]
Potato EVs	Scavenge free radicals; Downregulate MMPs; Weaken inflammatory response	120,000× *g*	Fresh; Whole	PBS	HaCaT	60 nm	in vitro	Anti-inflammatory	[57]
Cherry EVs	Antioxidant activity	/	/	/	/	/	/	Anti-photoaging	[98]
EC-EVs	Inhibit NOX/8-OHdG/p16; Upregulate HSP70	100,000× *g*	Fresh; Whole	/	Human epidermal keratinocytes	137.6 nm	in vitro + in vivo	Anti-photoaging	[101]
Apple callus EVs	Promote COL1A1/FBN1 synthesis	10,000× *g*	Fresh; Malus domestica	PBS	HDF, HaCaT	139.4 nm	in vitro	ECM synthesis enhancement	[107]
ADNPs	Activates Nrf2; Reduces ROS; Inhibits β-gal and SASP	150,000× *g*	Fresh; Gel or rind	PBS; Microneedle	HaCaT	190 nm; 160 nm	in vitro + in vivo	Anti-photoaging	[99]
GENs	Reduce ROS/SA-β-gal; Downregulate IL-6 and IL-1β; Downregulate p53/p21/MMP1	100,000× *g*	Fresh; parts without skin and seeds	PBS	HaCaT	178.2 nm	in vitro + in vivo	Anti-photoaging	[100]
LELNs	Regulate inflammation via miR166; Improve collagen preservation	150,000× *g*	Dry; Flowers	PBS	Fibroblasts	160.1 nm	in vitro + in vivo	Anti-photoaging	[103]
Cm-callus EVs	Promote collagen synthesis, angiogenesis, inhibit MMP1, anti-photoaging	150,000× *g*	Fresh; Leaves callus	/	Fibroblasts	136.6 nm	in vitro	Anti-photoaging	[85]

ROS, reactive oxygen species; SOD, superoxide dismutase; NF-κB, nuclear factor kappa-light-chain-enhancer of activated B cells; HDF, human dermal fibroblasts; HaCaT, human immortalized keratinocyte cell line; SA-β-gal, senescence-associated beta-galactosidase; IL-6, interleukin-6; MMPs, matrix metalloproteinases; NOX, NADPH oxidase; 8-OHdG, 8-hydroxy-2′-deoxyguanosine; p16/p21, cyclin-dependent kinase inhibitors; HSP70, heat shock protein 70; COL1A1, collagen type i alpha 1 chain; FBN1, fibrillin-1; Nrf2, nuclear factor erythroid 2-related factor 2; SASP, senescence-associated secretory phenotype; ECM, extracellular matrix.

## 5. Conclusions

UV-induced photoaging and wound healing represent the two predominant focuses in traumatic skin frontier. Wound healing is a complex process involving a variety of cells and molecules, and a slow healing rate seriously impairs the physical and mental health of patients. Photoaging leads to DNA damage and ROS production, which initiates an inflammatory response and changes cell structure and function. EVs derived from natural biological resources, particularly Ti-EVs and PDEVs, have emerged as promising candidates for cell-free therapies in skin regeneration and anti-photoaging. Both Ti-EVs and PDEVs can enhance the proliferation and migration of keratinocytes and dermal fibroblasts, stimulate angiogenesis, regulate inflammation, as well as promote ECM remodeling during wound healing. During the process of photodamage repair, natural-resource-derived EVs can promote collagen synthesis, enhance antioxidant capacity, and alleviate cell aging. Compared with cell-medium- and body-fluid-derived EVs, Ti-EVs offer a more physiologically relevant molecular repertoire that mirrors the multicellular and ECM-rich tissue microenvironment, potentially conferring superior bioactivity and tissue-specific repair signals. PDEVs, on the other hand, provide a scalable, low-cost source with unique plant-derived antioxidants and low immunogenicity.

## 6. Future Challenges

Natural-resource-derived EVs represent promising strategies for skin repair, yet their practical application is severely limited by a series of challenges. For human-tissue-derived EVs, limited tissue sources and ethical considerations constrain their preparation and application. With the development of gene-edited xenotransplantation, standardized, pathogen-safe, and low-immunogenic animal tissues are becoming more accessible. From an industrial perspective, this provides a reliable upstream supply chain and regulatory foundation for the clinical translation of animal-tissue-derived EVs. However, animal-tissue-derived EVs may include potential immunogenicity and residual pathogen risks, requiring strict safety evaluations. For plant-tissue-derived EVs, standardized cultivation and raw material specifications must be paired to ensure batch consistency.

Apart from the sample resources, there still exist some other problems that need to be resolved before the natural-resource-derived EVs can be widely applied in skin repair. Firstly, there is no generally accepted isolation and purification method suitable for the large-scale production of EVs from complex natural resources; thus, it is extremely necessary to establish a standardized and large-scale production protocol while warranting the repeatability of EV bioactivity. Translating naturally derived EVs from bench to bedside will require coordinated advances across sample collection, extraction and isolation, characterization, activity and safety assessment, and rigorous clinical evaluation. The standardization of sample collection, sample extraction and EV isolation is necessary for comparing the efficacy of different EV preparations and the results across studies. Secondly, despite the considerable activities of natural-resource-derived EVs in skin repair, there is a lack of acknowledged application forms and corresponding details for each kind of EV. Thirdly, the functional activities of Ti-EVs and PDEVs in skin repair received extensive recognition, but the in-depth molecular mechanism underlying which EVs exhibit biological functionality on wound healing and photoaging repair still remains elusive. Fourth, critical safety considerations, including donor screening criteria for Ti-EVs and tumorigenic risk assessments for pathological-tissue-derived EVs remain unaddressed. Addressing these interrelated challenges will require coordinated efforts in developing standardized manufacturing workflows and establishing preclinical models with clinically relevant endpoints. Lastly, clinical trials of natural-resource-derived EVs are still limited. Four registered studies based on PDEVs (NCT01668849, NCT04879810, NCT01294072, NCT03493984) were found, while three registered studies based on adipose-tissue-derived EVs (NCT06253975, NCT06444646, NCT05475418) were reported, and substantive data have yet to be published for any of these trials [6]. Despite both PDEVs and Ti-EVs having high potentials as novel therapeutic treatments, their incorporation into routine clinical use still requires more and deeper clinical trials to verify their effects and long-term safety in humans. These challenges mentioned above might be comprehensively addressed through interdisciplinary cooperation in the future, which can further provide constructive insights for developing EV-based therapeutic agents and driving their widespread application in regenerative medicine.

## Data Availability

No new data were created or analysed in this study. Data sharing is not applicable to this article.
